# Supplementary material for: Safety of changes in the use of noninvasive ventilation and high flow oxygen therapy on reintubation in a surgical intensive care unit: A retrospective cohort study
Source: PLoS One. 2021 Mar 22;16(3):e0249035. doi: 10.1371/journal.pone.0249035 (PMC7984629; doi:10.1371/journal.pone.0249035)
Supplement: S1 Table — FiO2: Fraction inspired of oxygen. NIV: Noninvasive ventilation. HFO: High-flow oxygenation. (DOCX) [file pone.0249035.s001.docx]

|  |  | All | P1 | P2 | *p* |
| --- | --- | --- | --- | --- | --- |
| NIV | **N** | 33 | 23 | 10 |  |
|  | **Days between extubation and NIV implementation** | 0.0 [0.0-1.0] | 0.0 [0.0-0.0] | 0.5 [0.0-1.5] | 0.221 |
|  | **Initial prescription:**  Session duration, h  Number of sessions per day  Total time per day, h | 3.0 [2.0-5.7]  1.5 [1.0-4.7]  7.5 [4.0-12.0] | 2.0 [2.0-5.2]  1.5 [1.0-4.2]  7.0 [4.5-13.0] | 3.5 [2.7-8.0]  1.5 [1.0-6.0]  8.0 [4.0-10.5] | 0.235  0.857  0.704 |
|  | **Inspiratory pressure support, cmH2O** | 10 [8-11] | 8 [8-12] | 10 [8-10] | 0.784 |
|  | **Expiratory positive pressure, cmH2O** | 5 [5-6] | 5 [5-6] | 6 [5-6] | **0.043** |
|  | **FiO2, %** | 40 [30-45] | 40 [30-45] | 37 [30-47] | 0.775 |
|  | **Therapy really performed:**  Number of days  Ratio Hours/Days therapy, h/d | 1 [1-3]  7.0 [4.0-10.0] | 1 [1-3]  7.0 [3.2-11.7] | 1 [1-2]  8.0 [4.0-9.2] | 0.603  0.787 |
|  | **Unplanned therapy termination**  Death  Reintubation  Intolerance  Switch for HFO | 14 (42.4)  1 (3.0)  11 (33.3)  1 (3.0)  1 (3.0) | 10 (43.5)  1 (4.3)  7 (30.4)  1 (4.3)  1 (4.3) | 4 (40.0)  0  4 (40.0)  0  0 | 0.646 |
| HFO | **N** | 49 | 25 | 24 |  |
|  | **Days between extubation and HFO implementation** | 0.0 [0.0-1.0] | 0.0 [0.0-2.0] | 0.0 [0.0-0.0] | **0.018** |
|  | **Initial prescription:**  Session duration, h  Number session by day  Total time by day, h | 19.0 [9.5-24.0]  1.0 [1.0-1.0]  20.0 [11.0-24.0] | 18.0 [4.5-24.0]  1.0 [1.0-1.0]  18.0 [7.5-24.0] | 20.0 [15.0-23.7]  1.0 [1.0-1.0]  20.0 [18.2-23.7] | 0.295  0.662  0.228 |
|  | **Debit, l/min** | 50 [50-60] | 50 [50-60] | 50 [50-60] | 0.283 |
|  | **FiO2, %** | 50 [40-50] | 50 [42-60] | 45 [32-50] | 0.071 |
|  | **Therapy really performed:**  Number of days  Ratio Hours/Days therapy, h/d | 2 [1-4]  18.0 [12.0-21.3] | 2 [1-4]  13.5 [7.0-18.7] | 2 [1-4]  20.9 [17.7-22.5] | 0.769  **0.001** |
|  | **Unplanned therapy termination**  Death  Reintubation  Intolerance | 14 (28.6)  0  14 (28.6)  0 | 9 (36.0)  0  9 (36.0)  0 | 5 (20.8)  0  5 (20.8)  0 | 0.094 |

**S1 Table. Characteristics of use and setting of noninvasive methods**

FiO2: Fraction inspired of oxygen. NIV: Noninvasive ventilation. HFO: High-flow oxygenation.
