# Supplementary material for: Safety of changes in the use of noninvasive ventilation and high flow oxygen therapy on reintubation in a surgical intensive care unit: A retrospective cohort study
Source: PLoS One. 2021 Mar 22;16(3):e0249035. doi: 10.1371/journal.pone.0249035 (PMC7984629; doi:10.1371/journal.pone.0249035)
Supplement: S2 Table — n (% of group). Odd ratio [95% Confidence Interval]: OR[95%IC]. BMI: Body mass index. COPD: Chronic obstructive pulmonary disease. OSA: Obstructive sleep apnea. ICU: Intensive care unit. ARDS: Acute respiratory distress syndrome. P/F: Arterial partial pressure of oxygen (PaO2 in mmHg)/Fraction inspired of oxygen (FiO2). a Reintubation within 7 days after extubation (excluding reintubation for surgery). (DOCX) [file pone.0249035.s002.docx]

| Characteristics | Reintubation within 7 days ^a^ (n=40) | Extubation success  (n=250) | Univariate  *p* | Multivariate regression OR[95%CI], *p* | |
| --- | --- | --- | --- | --- | --- |
| Male sex | 28 (70.0) | 33 (56.9) | 0.594 | - |  |
| Age, years | 66 [57-79] | 65 [50-73] | 0.128 | 1.01 [0.99-1.04] | 0.293 |
| BMI | 27.1 [22.8-32.9] | 26.7 [22.4-30.1] | 0.291 | - |  |
| SAPS II | 49 [41-57] | 48 [37-59] | 0.584 | - |  |
| Medical history  COPD  OSA  Arterial Hypertension  Coronary artery disease | 3 (7.5)  4 (10.0)  21 (52.5)  2 (5.0) | 17 (6.8)  15 (6.0)  109 (43.6)  24 (9.6) | 0.745  0.311  0.309  0.550 | -  -  -  - |  |
| Reason for ICU admission  Pneumonia  Acute pulmonary edema  ARDS  Coma  Shock | 2 (5.0)  0  0  17 (42.5)  8 (20.0) | 32 (12.8)  3 (1.2)  13 (5.2)  79 (31.6)  87 (34.8) | 0.192  1.000  0.227  0.205  0.071 | -  -  -  -  0.51 [0.19-1.23] | 0.153 |
| Postoperative admission  Postoperative day | 21 (52.5)  0 [0-0] | 170 (70.0)  0 [0-0] | **0.044**  0.622 | 0.50 [0.24-1.06]  - | 0.067 |
| P/F at admission | 269 [184-377] | 293 [169-401] | 0.571 | - |  |
| Extubation day | 5 [2-14] | 2 [1-7] | **0.013** | 1.04 [1.00-1.09] | 0.063 |
| P/F at extubation | 271 [208-326] | 289 [220-370] | 0.064 | 1.00 [0.99-1.00] | 0.211 |
| Phase 2 | 10 (25.0) | 99 (39.6) | 0.081 | 0.47 [0.18-1.09] | 0.093 |
| Preventive strategy | 9 (22.5) | 19 (7.6) | **0.007** | 1.44 [0.41-4.95] | 0.562 |
| Use of a noninvasive method  None  NIV used alone  HFO used alone  Combination | 22 (55.0)  5 (12.5)  6 (15.0)  7 (17.5) | 198 (82.5)  16 (6.7)  31 (12.9)  5 (2.0) | **<0.001**  Reference  0.065  0.266  **<0.001** | -  1.68 [0.42-5.73]  1.35 [0.39-4.14]  **6.70 [1.46-31.43]** | 0.104  -  0.433  0.617  **0.013** |

**S2 Table. Multivariate analysis of factors associated with reintubation within 7 days, integrating study phase.**

n (% of group). Odd ratio [95 % Confidence Interval]: OR[95%IC].

^a^ Reintubation within 7 days after extubation (excluding reintubation for surgery)

BMI: Body mass index. COPD: Chronic obstructive pulmonary disease. OSA: Obstructive sleep apnea. ICU: Intensive care unit. ARDS: Acute respiratory distress syndrome. P/F: Arterial partial pressure of oxygen (PaO_2_ in mmHg) / Fraction inspired of oxygen (FiO_2_).
