# Supplementary material for: Safety of changes in the use of noninvasive ventilation and high flow oxygen therapy on reintubation in a surgical intensive care unit: A retrospective cohort study
Source: PLoS One. 2021 Mar 22;16(3):e0249035. doi: 10.1371/journal.pone.0249035 (PMC7984629; doi:10.1371/journal.pone.0249035)
Supplement: S3 Table — n (% of group). Hazard ratio [95% Confidence Interval]: HR[95%IC]. -: Not available. BMI: Body mass index. COPD: Chronic obstructive pulmonary disease. OSA: Obstructive sleep apnea. ICU: Intensive care unit. P/F: Arterial partial pressure of oxygen (PaO2)/Fraction inspired of oxygen (FiO2). a Reintubation within 7 days after extubation (excluding reintubation for surgery). (DOCX) [file pone.0249035.s003.docx]

| Characteristics | Reintubation within 7 days ^a^ (n=40) | Extubation success  (n=250) | Univariable  HR | *p* | Multivariable model  HR[95%CI] *p* | |
| --- | --- | --- | --- | --- | --- | --- |
| Male sex | 28 (70.0) | 33 (56.9) | 1.25 | 0.524 |  |  |
| Age, years | 66 [57-79] | 65 [50-73] | - | 0.158 |  |  |
| BMI | 27.1 [22.8-32.9] | 26.7 [22.4-30.1] | - | 0.871 |  |  |
| SAPS II | 49 [41-57] | 48 [37-59] | - | 0.682 |  |  |
| Medical history  COPD  OSA  Arterial Hypertension  Coronary artery disease | 3 (7.5)  4 (10.0)  21 (52.5)  2 (5.0) | 17 (6.8)  15 (6.0)  109 (43.6)  24 (9.6) | 1.15  1.60  1.38  0.52 | 0.811  0.372  0.308  0.367 |  |  |
| Reason for ICU admission  Pneumonia  Acute pulmonary edema  ARDS  Shock | 2 (5.0)  0  0  8 (20.0) | 32 (12.8)  3 (1.2)  13 (5.2)  87 (34.8) | 0.37  0.00  0.00  0.48 | 0.176  0.997  0.996  0.067 | 0.54 [0.23-1.26] | 0.154 |
| Postoperative admission | 21 (52.5) | 170 (70.0) | 0.52 | **0.036** | 0.56 [0.30-1.07] | 0.080 |
| P/F at admission | 269 [178-386] | 293 [167-402] | - | 0.723 |  |  |
| Extubation day | 5 [2-14] | 2 [1-7] | **-** | **0.011** | 1.03 [0.99-1.06] | 0.122 |
| P/F at extubation | 271 [208-326] | 289 [220-370] | **-** | 0.060 | 1.00 [1.00-1.00] | 0.268 |
| Phase 2 | 10 (25.0) | 99 (39.6) | 0.52 | 0.074 | 0.47 [0.22-1.04] | 0.061 |
| Preventive strategy | 9 (22.5) | 19 (7.6) | 2.88 | **0.005** | 1.43 [0.55-3.69] | 0.463 |
| Use of a noninvasive method  None  NIV used alone  HFO used alone  Combination | 22 (55.0)  5 (12.5)  6 (15.0)  7 (17.5) | 198 (82.5)  16 (6.7)  31 (12.9)  5 (2.0) | Reference  2.55  1.60  7.64 | **0.001**  -  0.059  0.309  **0.001** | Reference  1.65 [0.55-4.90]  1.25 [0.44-3.58]  **4.37 [1.37-13.92]** | 0.109  -  0.371  0.667  **0.013** |

**S3 Table. Multivariable Cox analysis of factors associated with reintubation integrating study phase.**

n (% of group). Hazard ratio [95 % Confidence Interval]: HR[95%IC]. -: not available

^a^ Reintubation within 7 days after extubation (excluding reintubation for surgery)

BMI: Body mass index. COPD: Chronic obstructive pulmonary disease. OSA: Obstructive sleep apnea. ICU: Intensive care unit. P/F: Arterial partial pressure of oxygen (PaO_2_) / Fraction inspired of oxygen (FiO_2_).
