# Supplementary material for: Safety of changes in the use of noninvasive ventilation and high flow oxygen therapy on reintubation in a surgical intensive care unit: A retrospective cohort study
Source: PLoS One. 2021 Mar 22;16(3):e0249035. doi: 10.1371/journal.pone.0249035 (PMC7984629; doi:10.1371/journal.pone.0249035)
Supplement: S4 Table — No statistical comparisons were made due to the small number of patients. (DOCX) [file pone.0249035.s004.docx]

| Combination of NIV and HFO | All (n=12) | P1 (n=7) | P2 (n=5) |
| --- | --- | --- | --- |
| NIV used first | **2** | **2** | **0** |
| Indication of NIV  Curative  Preventive | 1  1 | 1  1 |  |
| Indication of HFO  Curative | 2 | 2 |  |
| HFO used first | **4** | **1** | **3** |
| Indication of HFO  Preventive | 4 | 1 | 3 |
| Indication of NIV  Curative | 4 | 1 | 3 |
| NIV/HFO started the same day | **6** | **4** | **2** |
| Indication  Curative  Preventive | 4  2 | 2  2 | 2  0 |

**S4 Table. Detail of combined use of NIV and HFO.**

No statistical comparisons were made due to the small number of patients.
