# Supplementary material for: Safety of changes in the use of noninvasive ventilation and high flow oxygen therapy on reintubation in a surgical intensive care unit: A retrospective cohort study
Source: PLoS One. 2021 Mar 22;16(3):e0249035. doi: 10.1371/journal.pone.0249035 (PMC7984629; doi:10.1371/journal.pone.0249035)
Supplement: Attachment — Submitted filename: PLOS1 review.docx [file pone.0249035.s006.docx]

Plos one review Criteria:

1. Primary scientific research:

- In the introduction, the authors wrote a study comparing high-flow nasal cannula (HFO) vs non-invasive ventilation (NIV) have been not been performed, but there is a number of qualitative retrospective studies published. Also, there have been two multi-center prospective randomized controlled studies comparing the two treatments for re-intubation:
  - Post-surgery: High-Flow Nasal Oxygen vs Noninvasive Positive Airway Pressure in Hypoxemic Patients After Cardiothoracic Surgery: A Randomized Clinical Trial. Barrucand et al, BiPOP Study Group. JAMA. 2015 Jun 16; 313(23):2331-9
  - Medical ICU: Effect of Postextubation High-Flow Nasal Cannula vs Noninvasive Ventilation on Reintubation and Postextubation Respiratory Failure in High-Risk Patients: A Randomized Clinical Trial. Hernández G, et.al. JAMA. 2016 Oct 18; 316(15):1565-1574.
- The two randomized trials when pooled together in a meta-analysis found no difference in re-intubation rates. Xu et al. High-flow nasal cannula in adults with acute respiratory failure and after extubation: a systematic review and meta-analysis. *Respir Res*. 2018;19(1):202. Published 2018 Oct 16. doi:10.1186/s12931-018-0908-7

2. Results not reported elsewhere:

It does not appear that they have published this elsewhere. However, there are similar studies like this published.

3. Experiments, statistics, and other analyses are performed to high technical standard and described in sufficient detail

- This is a pre/post design of single retrospective cohort for physician education on High-Flow nasal cannula (HFO). It seems like the purpose is a quality improvement study to see if change of practice has adversely affected patient outcomes. Endpoint is reintubation within 48 hours. The authors imply during April 2016 to 2017 that this was a training period or “wash-out” and so do not include data during this period. One year is a bit long to explain training and no further details if new protocols were instituted. Therefore, the difference in Phase 2 and Phase 1 may have multiple confounding factors that explains less re-intubation rates.
- They also do not include patients who were on conventional treatment of nasal cannula or simple face mask. If this institution only uses high-flow nasal cannula vs NIV for post-op, this wasn’t entirely clear. Also, this paper was not convincing of an advantage of a higher cost modality over the conventional therapy of nasal cannula (I.e. no description or risk factors which patients seem to most benefit with HFO.)
- Including all consecutive patients admitted to ICU is a good design, but the methods do not describe why patients were on NIV vs conventional method, therefore an issue with selection bias. They do not describe a criteria or protocol, but rather is this is individual physician-decision driven.
- The driving cause for reintubation in Phase 1 was neurologic failure and shock (in addition to respiratory failure). NIV does not cause neuro failure or shock, so it appears a confounding severity was not sufficiently controlled for in the analysis (I.e. factors causing hypercapnia such as sedation or differences in fluid status).
- “Reintubation delay” implies that there was a missed opportunity such that reintubation could have been or should have been reintubated earlier. How did you determine “reintubation delay”? was not clearly defined in the methods. If the authors wanted to just note the differences, consider a different term such as “time to reintubation”.
- The category of combination of non-invasive method is very heterogenous. The study design doesn’t clearly state how was this done or decided. (i.e. was changed from HFO to NIV or NIV to HFO and why?) The main issue with the study design is confounding by indication and severity. It is likely clinical indication was the driver for the selection of the treatment, that more severe patients were placed on NIV rather than HFO.

4. Conclusions are presented in appropriate fashion and supported by the data
   a. Lower P2 reintubation rates seem to be due to selection bias. To say more HFO use is better than NIV and conventional therapy in preventing acute respiratory failure is a bit of a leap, especially since shock and neurologic failure are contributing to the respiratory failure. There was generally more HFO use due to education on this treatment. Since it is a newer treatment, physicians may have been more apt to use the more established NIV treatment that they are more familiar with than the newer technology or wish to deliver more positive pressure due to concerns of severe respiratory failure signs.
   b. Table 3 is confusing: column reintubation at 48 hour is N=24, but one of the sections below “use of a noninvasive method” doesn’t match (22+5+6+7=40 which is the reintubation at 7 days). For “extubation success”, at the top n=266, so this section also do not add up (198+16+31+5=250). The row of “phase 2” is alone without a Phase 1 row.
   c. The authors did a fair job in capturing severity of disease between phase 1 and phase 2, but not between HFO and NIV patients.
   d. Would be helpful to include data on conventional therapy during the time periods with comparison as controls
   e. This is not a non-inferiority study so can only be hypothesis generating, which the authors are aware in their discussion, but the conclusion may be misleading when they state HFO was used more in P2 and therefore had less re-intubation rates. Also, absence of statistical significant difference does not infer therapies have similar effects or efficacy, especially since no control and no uniformity or protocol on how these therapies were applies. Propensity matching may help with some unaccounted confounding variables. Neither HFNO or NIV post-extubation is gold-standard post-extubation, so comparison with conventional therapy would have been helpful in determining if the reported reintubation rates are expected.
5. Article is intelligible and written in standard English
   a. Some awkward phrasing. Needs refinement in editing and revision throughout.
6. Research meets all applicable standards for the ethics of experimentation and research integrity- yes
7. Article adheres to appropriate reporting guidelines and community standards for data availability – yes if authors are willing to have data sharing available
