# Supplementary material for: Safety of changes in the use of noninvasive ventilation and high flow oxygen therapy on reintubation in a surgical intensive care unit: A retrospective cohort study
Source: PLoS One. 2021 Mar 22;16(3):e0249035. doi: 10.1371/journal.pone.0249035 (PMC7984629; doi:10.1371/journal.pone.0249035)
Supplement: Attachment — Submitted filename: Response_to_review_ARVENIO.docx [file pone.0249035.s007.docx]

**Corresponding author:**

Stanislas ABRARD M.D.

Département d’Anesthésie Réanimation

Hôpital Édouard Herriot

Hospices Civils de Lyon

5 place d’Arsonval

69437 Lyon CEDEX 03

e-mail : stanislas.abrard@chu-lyon.fr

Phone: + 33 4 72 11 69 44

Dear Editor,

I am pleased to submit this revised version of our manuscript (Ref: PONE-S-20-37260) entitled “Effect of changes in the use of noninvasive ventilation and high flow oxygen therapy on reintubation in a surgical intensive care unit. A Retrospective Cohort Study.” for possible publication in Plos One.

You will find below the point-by-point response to reviewers' comments. The changes are reported in red in the revised text.

We, authors of PONE-S-20-37260, thank you for your time and consideration. Reviewers’ comments helped us revise the manuscript, and we believe that content and clarity are now improved in this revised version.

Sincerely yours,

Stanislas Abrard

**Academic editors’ comments:**

The authors should note that one of the reviewers felt there was extensive need for editing, specifically asking for more details on study design and results.  I would also add that is unclear what processes occurred between phase 1 and phase 2 that resulted in more HFO2 use: education amongst physicians? education of respiratory therapists?  New protocols/policies?  These need to be spelled out.

**Thank you for your relevant comments. Clarifications about the interphase have been added in the paragraph “Material and Methods”, lines 102 to 110.**

“Between the two phases, physicians of the unit received peer education and training on the benefits and use of HFO and gained experience of it. No other changes in the respiratory management of patients was implemented, particularly no change in protocols. A period of one year was chosen between the 2 phases as changing practices is a long-term process. A sufficient time was needed for peer-to-peer sharing of information and experience as well as having historical perspective on the first use of HFO by new users.” **In France, respiratory management depends on the physicians and not on the respiratory therapists.**

Finally, as the reviewer pointed out there has been randomized studies showing no difference in outcomes between NIV and HFO2; the authors need to discuss why there results are different and whether clinically relevant given randomized trial data.

**To our knowledge, randomized studies showed no difference** **in outcomes between NIV and HFO. As indicated in the “Discussion”, our results are concordant with these randomized studies.**

***Line 278: “In line with previous published studies, the occurrence of reintubation was not specifically associated with one or the other method in our study.”***

**The initial formulation of the conclusion in the abstract has been clarified: “The earlier implementation of noninvasive methods and the increased use of HFO between the 2 phases was associated with a lower extubation failure rate at 48 hours.”**

**In the ethics statement in the manuscript and in the online submission form, please provide additional information about the patient records used in your retrospective study, including: a) whether all data were fully anonymized before you accessed them and/or whether the IRB or ethics committee waived the requirement for informed consent; If patients provided informed written consent to have data from their medical records used in research, please include this information.**

**Patients’ records were not anonymized before we accessed it. We have proceeded to the database anonymization before the analysis. As required by the French law for observational studies (Toulouse E, Masseguin C, Lafont B, et al. French legal approach to clinical research. Anaesth Crit Care Pain Med. 2018), patients were advised during their hospital stay that their data could be used retrospectively for clinical research purpose. A written informed consent was not required for observational retrospective studies, but patients could refuse to use their data upon request.**

**b) the date range (month and year) during which patients' medical records were accessed;**

**April to July 2020. The information has been added line 108.**

**c) the source of the medical records analyzed in this work (e.g. hospital, institution or medical center name).**

**University Hospital of Angers, Angers, France**

**These information have been added in the** **manuscript, line 108, and in the online submission form.**

**We note that you have indicated that data from this study are available upon request. PLOS only allows data to be available upon request if there are legal or ethical restrictions on sharing data publicly.**

**In your revised cover letter, please address the following prompts:**

**a) If there are ethical or legal restrictions on sharing a de-identified data set, please explain them in detail (e.g., data contain potentially identifying or sensitive patient information) and who has imposed them (e.g., an ethics committee). Please also provide contact information for a data access committee, ethics committee, or other institutional body to which data requests may be sent.**

**b) If there are no restrictions, please upload the minimal anonymized data set necessary to replicate your study findings as either Supporting Information files or to a stable, public repository and provide us with the relevant URLs, DOIs, or accession numbers. Please see http://www.bmj.com/content/340/bmj.c181.long for guidelines on how to de-identify and prepare clinical data for publication. For a list of acceptable repositories, please see http://journals.plos.org/plosone/s/data-availability#loc-recommended-repositories.**

**We will update your Data Availability statement on your behalf to reflect the information you provide.**

**There are no restrictions. We have uploaded the anonymized database as Supplement Material. The cover letter has been revised.**

**Reviewers’ comments:**

**Reviewer #2:**

**Data in the abstract and data in the tables don't align or just isn't clear. For example, in the abstract the HFO use in P2 is 24 with 83% and in Table 1 P2 has 24 with percent of 22%.**

**Thank you for this very relevant comment. Data in the abstract and in Table 1 were not aligned because the percentage was calculated on the entire cohort for Table 1 and only on patients with noninvasive ventilation for the abstract. The abstract has been revised.**

**In looking at the table it looks like the 2nd time period had less HFO use (greater percentage is true) and then had a decreased reintubation rate at 48 hour. They stated that there was a increase in education between the 2 time groups about HFO and while statistically significant the clinical significant of 1 patient is questionable.**

**Indeed, unfortunately, there were less patients who met our inclusion criteria in phase 2, without obvious explanation. However, we decided to maintain to compare these two phases as this was relevant for us according to the changes in the use of NIV and HFO in our unit. In addition, this change of use was confirmed by the higher rate of HFO use, which was almost twice higher in P2 than in P1 (22.0% vs 13.8%, p=0.039), which seems clinically significant.**

**In addition about 30% of the patient in the study are not postoperative patients. If want to make an assessment about HFO in SICU patient population it should all be surgical or at least define where these nonoperative patients come from (i.e. are they non-operative trauma, head injury, etc.)**

**Indeed, almost 30% were not postoperative patients. However, they were usual patients hospitalized in surgical ICUs, with 38 trauma patients + 25 patients with head injury. As requested, the origin of non-surgical patients has been clarified in Table 1.**

**There are several questionable typing errors of the p being reported at p<.15 on line 143, which I'm assuming should be p<0.05?**

**There were no typing errors about the p-value cut-off in the selection process of candidate predictor variables. As indicated in line 143, variables with p-value < 0.15 in univariable analysis were integrated into the multivariable model.**

**To avoid confusion with the p-value cut off indicating the statistical significance, we have added the following sentence at the end of Methods paragraph:**

***“A p-value <0.05 was considered to indicate statistical significance.”***

**Reviewer #1:**

**1.	Primary scientific research:**

**•	In the introduction, the authors wrote a study comparing high-flow nasal cannula (HFO) vs non-invasive ventilation (NIV) have been not been performed, but there is a number of qualitative retrospective studies published. Also, there have been two multi-center prospective randomized controlled studies comparing the two treatments for re-intubation:**

**o	Post-surgery: High-Flow Nasal Oxygen vs Noninvasive Positive Airway Pressure in Hypoxemic Patients After Cardiothoracic Surgery: A Randomized Clinical Trial. Barrucand et al, BiPOP Study Group. JAMA. 2015 Jun 16; 313(23):2331-9**

**o	Medical ICU: Effect of Postextubation High-Flow Nasal Cannula vs Noninvasive Ventilation on Reintubation and Postextubation Respiratory Failure in High-Risk Patients: A Randomized Clinical Trial. Hernández G, et.al. JAMA. 2016 Oct 18; 316(15):1565-1574.**

**•	The two randomized trials when pooled together in a meta-analysis found no difference in re-intubation rates. Xu et al. High-flow nasal cannula in adults with acute respiratory failure and after extubation: a systematic review and meta-analysis. Respir Res. 2018;19(1):202. Published 2018 Oct 16. doi:10.1186/s12931-018-0908-7**

**Thank you for this remark. This part of the introduction was indeed poorly formulated. The introduction has been modified, taking into account your remarks and references. We also clarified that the comparison of NIV and OHD was not the primary objective of this study.**

**2.	Results not reported elsewhere:**

**It does not appear that they have published this elsewhere. However, there are similar studies like this published.**

**To our knowledge, this study is the only one aimed to describe the evolution of the use of noninvasive methods and the impact on clinical outcomes in a real-life setting. Many studies that focused on the post-operative use of noninvasive methods were limited to the comparison of NIV and HFO. In addition, few have included patients for whom these techniques were not used. Lastly, in our study, physicians were free to choose the strategy they thought most appropriate. No protocol modifications were made. The changes in the use of non-invasive methods are, therefore, likely to be spontaneously observed in any ICU.**

**3.	Experiments, statistics, and other analyses are performed to high technical standard and described in sufficient detail**

**-	This is a pre/post design of single retrospective cohort for physician education on High-Flow nasal cannula (HFO). It seems like the purpose is a quality improvement study to see if change of practice has adversely affected patient outcomes. Endpoint is reintubation within 48 hours. The authors imply during April 2016 to 2017 that this was a training period or “wash-out” and so do not include data during this period. One year is a bit long to explain training and no further details if new protocols were instituted. Therefore, the difference in Phase 2 and Phase 1 may have multiple confounding factors that explains less re-intubation rates.**

**As indicated in answer to the Editors’ remark, clarifications about this “wash-out period” have been added in the paragraph “Material and Methods”, lines 102 to 110:**

“Between the two phases, physicians of the unit received peer education and training on the benefits and use of HFO and gained experience of it. No other changes in the respiratory management of patients was implemented, particularly no change in protocols. A period of one year was chosen between the 2 phases as changing practices is a long-term process. A sufficient time was needed for peer-to-peer sharing of information and experience as well as having historical perspective on the first use of HFO by new users.**”**

**Indeed, we believe that changing practices is a long process. A sufficient time was needed for peer-to-peer sharing of information and experience as well as having a historical perspective on the first use of HFO by new users.**

**Multiple confounding factors were taken into account in the multivariable analysis, but the retrospective design does not allow to totally exclude a persistent bias.**

**-	They also do not include patients who were on conventional treatment of nasal cannula or simple face mask. If this institution only uses high-flow nasal cannula vs NIV for post-op, this wasn’t entirely clear. Also, this paper was not convincing of an advantage of a higher cost modality over the conventional therapy of nasal cannula (I.e. no description or risk factors which patients seem to most benefit with HFO.)**

**Thank you for allowing us to clarify this point. As indicated in the “Participants” paragraph in “Methods”: “All adult patients (age≥18 years) hospitalized in the intensive care unit, intubated, mechanically ventilated for a duration > one day and extubated alive during the two studied phases were included in our cohort.” This means that patients who were on the conventional treatment of nasal cannula or simple face mask (i.e., conventional oxygen therapy) were included in the study and in the analysis. As indicated Table 1, 75.9% of patients were treated by conventional oxygen therapy (77.3% in P1 and 73.4% in P2, p=0.480).**

**The economic analysis was not planned in this work in order to avoid confusion and to be concise.**

**-	Including all consecutive patients admitted to ICU is a good design, but the methods do not describe why patients were on NIV vs conventional method, therefore an issue with selection bias. They do not describe a criteria or protocol, but rather is this is individual physician-decision driven.**

**As explained in the previous question, the use of NIV/HFO was not a criterion for inclusion. We believe the retrospective design reflects the real-life in this study. The decision to initiate NIV/HFO is individual physician-decision driven. Indication of initiation of noninvasive methods by the physician was mentioned in the medical case. We provided this information Table 1 (curative = treat acute respiratory failure / preventive = prevent acute respiratory failure).**

**-	The driving cause for reintubation in Phase 1 was neurologic failure and shock (in addition to respiratory failure). NIV does not cause neuro failure or shock, so it appears a confounding severity was not sufficiently controlled for in the analysis (I.e. factors causing hypercapnia such as sedation or differences in fluid status).**

**Sedation and fluid status were not registered in our study. Patients severity was registered through the SAPS II and P/F at admission and extubation. These variables were taken into account in multivariable when appropriate (p<0.15). Your important remark has been added to the Discussion (line 267-269).**

**-	“Reintubation delay” implies that there was a missed opportunity such that reintubation could have been or should have been reintubated earlier. How did you determine “reintubation delay”? was not clearly defined in the methods. If the authors wanted to just note the differences, consider a different term such as “time to reintubation”.**

**Thanks for this relevant comment. The term “Reintubation delay” has been changed to “Time to reintubation” all along the entire manuscript.**

**-	The category of combination of non-invasive method is very heterogenous. The study design doesn’t clearly state how was this done or decided. (i.e. was changed from HFO to NIV or NIV to HFO and why?) The main issue with the study design is confounding by indication and severity. It is likely clinical indication was the driver for the selection of the treatment, that more severe patients were placed on NIV rather than HFO.**

**Details about the combination of NIV and HFO have been added in Supplement materials. Regrettably, the small number of patients does not allow to conclude about the combination of NIV and HFO. Literature is lacking about this question. Our results highlight the importance of further studies on this issue.**

**4.	Conclusions are presented in appropriate fashion and supported by the data**

**a.	Lower P2 reintubation rates seem to be due to selection bias. To say more HFO use is better than NIV and conventional therapy in preventing acute respiratory failure is a bit of a leap, especially since shock and neurologic failure are contributing to the respiratory failure. There was generally more HFO use due to education on this treatment. Since it is a newer treatment, physicians may have been more apt to use the more established NIV treatment that they are more familiar with than the newer technology or wish to deliver more positive pressure due to concerns of severe respiratory failure signs.**

**We understand your question. However, as explained above, we believe that the fact that all patients mechanically ventilated >24 hours were included in our study prevents, at least in part, a selection bias. Moreover, our conclusion is not to use less NIV (and there was no variation in the rate of use of NIV between the Phases) but that an earlier implementation of noninvasive method and an increased use of HFO is better in preventing acute respiratory (than not to do it). In addition, no significant difference in reintubation rate between NIV/HFO was found in our study.**

**Finally, we think that the long “wash-out” period avoids the possible effect of increasing the use of HFO due to the novelty of this treatment.**

**b.	Table 3 is confusing: column reintubation at 48 hour is N=24, but one of the sections below “use of a noninvasive method” doesn’t match (22+5+6+7=40 which is the reintubation at 7 days). For “extubation success”, at the top n=266, so this section also do not add up (198+16+31+5=250). The row of “phase 2” is alone without a Phase 1 row.**

**Thank you for reporting this error. Data of reintubation at 7 days were reported in place of reintubation at 48h. These mistakes have been corrected, and all tables reviewed.**

**By definition, patients not in the Phase 2 were in Phase 1. This is the reason why there was no line “Phase 1” in Table 3.**

**c.	The authors did a fair job in capturing severity of disease between phase 1 and phase 2, but not between HFO and NIV patients.**

**The comparison between characteristics of NIV and HFO patients was not presented because of concision. Data about patient’s severity were taking into account multivariable analysis.**

**Characteristics of NIV and HFO patients are presented below. We observe that patients treated by HFO were significantly younger, free to OSA, presented more frequently a comatose on admission, extubated later, and had a lower BMI. Regrettably, the number of patients in each group was too small to validate the multivariable analysis.**

| Characteristics | NIV alone (n=21) | HFO alone (n=37) | *p* |
| --- | --- | --- | --- |
| Male sex | 11 (52.4) | 22 (59.5) | 0.783 |
| Age, years | 74 [64-80] | 63 [47-73] | 0.009 |
| BMI | 32.2 [26.8-43.2] | 27.6 [23.8-30.3] | 0.030 |
| SAPS II | 49 [39-59] | 47 [39-61] | 0.871 |
| Medical history  COPD  OSA  Arterial Hypertension  Coronary artery disease | 3 (14.3)  9 (42.9)  13 (61.9)  1 (4.8) | 1 (2.7)  0 (0)  15 (40.5)  1 (2.7) | 0.130  <0.001  0.172  1.000 |
| Reason for ICU admission  Pneumonia  Acute pulmonary edema  ARDS  Coma  Shock | 7 (33.3)  0 (0)  2 (9.5)  3 (14.3)  6 (28.6) | 5 (13.5)  1 (2.7)  3 (8.1)  15 (40.5)  14 (37.8) | 0.097  1.000  1.000  0.044  0.572 |
| Postoperative admission  Postoperative day | 11 (52.4)  0 [0-3] | 28 (75.7)  0 [0-0] | 0.086  0.508 |
| P/F at admission | 193 [150-267] | 245 [178-297] | 0.369 |
| Extubation day | 2 [1-6] | 8 [2-12] | 0.003 |
| P/F at extubation | 232 [182-281] | 222 [162-277] | 0.467 |
| Reintubation within 48 hours ^a^, % | 2 (9.5) | 2 (5.4) | 0.615 |
| Reintubation within 7 days ^b^, % | 5 (23.8) | 6 (16.2) | 0.504 |

^a^ Reintubation within 48 hours after extubation (excluding reintubation for surgery)

^b^ Reintubation within 7 days after extubation (excluding reintubation for surgery)

**d.	Would be helpful to include data on conventional therapy during the time periods with comparison as controls**

**Characteristics of patients treated with conventional oxygen therapy during the 2 phases are presented below as control.**

| Phase | 1 | | | *2* | | |
| --- | --- | --- | --- | --- | --- | --- |
| Sub-group | NIV and/or HFO | Conventional oxygen therapy | *p* | NIV and/or HFO | Conventional oxygen therapy | *p* |
| N | 41 | 140 | *-* | 29 | 80 | *-* |
| Male sex | 26 (63.4) | 95 (67.9) | 0.706 | 16 (55.2) | 53 (66.3) | 0.369 |
| Age, years | 70 [52-78] | 64 [51-75] | 0.112 | 67 [51-73] | 62 [44-72] | 0.305 |
| BMI | 27.4 [24.3-32.2] | 26.7 [22.3-29.8] | 0.366 | 30.1 [24.1-38.2] | 26.1 [21.8-29.9] | **0.004** |
| SAPS II | 48 [36-63] | 48 [37-58] | 0.655 | 50 [42-59] | 47 [39-58] | 0.567 |
| Medical history  COPD  OSA  Arterial Hypertension  Coronary artery disease | 4 (9.8)  6 (14.6)  20 (48.8)  2 (4.9) | 6 (4.3)  6 (4.3)  64 (45.7)  20 (14.3) | 0.237  **0.030**  0.859  0.171 | 3 (10.3)  4 (13.8)  14 (48.3)  2 (6.9) | 7 (8.8)  3 (3.8)  32 (40.0)  2 (2.5) | 0.724  0.079  0.512  0.287 |
| Reason for ICU admission  Pneumonia  Acute pulmonary edema  ARDS  Coma  Shock | 8 (19.5)  2 (4.9)  2 (4.9)  11 (26.8)  9 (22.0) | 11 (7.9)  1 (0.7)  2 (1.4)  49 (35.0)  34 (24.3) | **0.043**  0.129  0.221  0.353  0.837 | 6 (20.7)  0  3 (10.3)  9 (31.0)  14 (48.3) | 9 (11.3)  0  6 (7.5)  27 (33.8)  38 (47.5) | 0.220  -  0.697  1.000  1.000 |
| Postoperative admission | 28 (68.3) | 96 (68.6) | 1.000 | 18 (62.1) | 54 (67.5) | 0.650 |
| P/F at admission | 245 [161-381] | 308 [179-420] | 0.154 | 180 [140-278] | 310 [210-406] | **<0.001** |
| Extubation day | 7 [2-9] | 2 [1-7] | **0.005** | 5 [2-14] | 2 [1-6] | **0.006** |
| P/F at extubation | 226 [180-279] | 312 [228-383] | **<0.001** | 214 [142-275] | 304 [247-373] | **<0.001** |
| Reintubation within 48 hours ^a^, % | 7 (17.1) | 13 (9.3) | 0.166 | 2 (6.9) | 2 (2.5) | 0.283 |
| Reintubation within 7 days ^b^, % | 11 (26.8) | 19 (13.6) | 0.056 | 7 (24.1) | 3 (3.8) | **0.003** |

**We can see that differences in patients’ characteristics between the 2 phases are small. Globally, patients treated by noninvasive methods were more severe than patients treated by conventional oxygen therapy. Reintubation rates at 7 days tended to be significantly higher in P1 and were significantly higher in P2 for patients treated by noninvasive methods. This effect can be explained by patient severity differences. In multivariable models presented in our study, after adjustment on the severity, the use of noninvasive methods was not associated with reintubation.**

**To our opinion, this table does not provide essential elements for understanding the study. We propose not to add it to the manuscript.**

**e.	This is not a non-inferiority study so can only be hypothesis generating, which the authors are aware in their discussion, but the conclusion may be misleading when they state HFO was used more in P2 and therefore had less re-intubation rates. Also, absence of statistical significant difference does not infer therapies have similar effects or efficacy, especially since no control and no uniformity or protocol on how these therapies were applies. Propensity matching may help with some unaccounted confounding variables. Neither HFNO or NIV post-extubation is gold-standard post-extubation, so comparison with conventional therapy would have been helpful in determining if the reported reintubation rates are expected.**

**Thank for this relevant comment. Particular attention has been paid to the correction of the discussion and the conclusion. The use of term “not significantly different” has been preferred to “similar.**

**A comparison with conventional therapy is presented above.**

**5.	Article is intelligible and written in standard English**

**a.	Some awkward phrasing. Needs refinement in editing and revision throughout.**

**Correction of grammatical errors and English improvement were carried done by a native English speaker.**
