# Supplementary material for: Safety of changes in the use of noninvasive ventilation and high flow oxygen therapy on reintubation in a surgical intensive care unit: A retrospective cohort study
Source: PLoS One. 2021 Mar 22;16(3):e0249035. doi: 10.1371/journal.pone.0249035 (PMC7984629; doi:10.1371/journal.pone.0249035)
Supplement: Attachment — Submitted filename: Response_to_review2_ARVENIO.docx [file pone.0249035.s009.docx]

**Corresponding author:**

Stanislas ABRARD M.D.

Département d’Anesthésie Réanimation

Hôpital Édouard Herriot

Hospices Civils de Lyon

5 place d’Arsonval

69437 Lyon CEDEX 03

e-mail : stanislas.abrard@chu-lyon.fr

Phone: + 33 4 72 11 69 44

Dear Editor,

I am pleased to submit this revised version of our manuscript (Ref: PONE-S-20-37260) entitled “Effect of changes in the use of noninvasive ventilation and high flow oxygen therapy on reintubation in a surgical intensive care unit. A Retrospective Cohort Study.” for possible publication in Plos One.

You will find below the point-by-point response to reviewers' comments. The changes are reported in red in the revised text.

We, authors of PONE-S-20-37260, thank you for your time and consideration. Reviewers’ comments helped us revise the manuscript, and we believe that content and clarity are now improved in this revised version. An Editing Service edited this manuscript.

Sincerely yours,

Stanislas Abrard

**Reviewer #1 comments:**

1.	There needs further revision for clarity of thought and free of grammatical errors. for example, in line 59, "pathophysiology" is not used correctly here. It is a term to describe the physiologic mechanism behind symptoms so should not be used to describe a list of clinical manifestations or symptoms. there should also be a "and" before cardiac dysfunction in line 60.

An Editing Service edited this manuscript. We replaced « pathophysiology » in line 59 by « causes ».

In line 67, the use of the word "curative" is also odd because NIV doesn't "cure" tracheal reintubation, since it is a supportive measure to bridge until the underlying process is addressed so should be referred to as "preventative".

Thank you for allowing us to clarify this point. Of course, the NIV does not treat tracheal intubation. We wanted to differentiate between two situations well described by the guidelines (Official ERS/ATS clinical practice guidelines: noninvasive ventilation for acute respiratory failure. Bram Rochwerg *et al*. Eur Resp J 2017): NIV for treatment of ARF in prevention of reintubation (patient with ARF criteria) named “curative” NIV in our previous manuscript and NIV for prevent post-extubation ARF (patient without AFR criteria) named “preventive” NIV in our previous manuscript. To improve clarity of our manuscript, in the revised version we named “prophylactic” NIV for prevent post-extubation ARF (patient without AFR criteria) and “supportive treatment by NIV” NIV for treatment of ARF in prevention of reintubation (patient with ARF criteria) to bridge until the underlying process.

In line 69, the phrase "a more recent method" is unclear and refers to that is a more recent developed technology compared to NIV but I think only a reader who would know that context would understand this, so instead of "recent method" should be changed to "a more recently developed technology". But that sentence doesn't clearly distinguish the difference between NIV and HFO.

This term has been changed and precisions have been added.

In lines 83 & 84, connect the reason to your unit's change in practice more clearly to the benefits as described in lines 73. Also, it would be more clear to define specific worsening outcomes in line 79, because patient safety should then be addressed as part of your primary objectives and results. In light of the two meta-analyses and studies of prospective randomized controlled studies, at the most your retrospective study can show no worsening of patients but would not be able to prove HFO is superior.

The benefits described in the introduction have been more clearly linked to the reason of our unit practice changes.

The introduction and discussion paragraphs have been greatly revised regarding patient safety during this change in practice and no worsening.

In line 109, this sentence "we proceeded to anonymization of database before data analysis" is awkwardly phrased and needs re-writing.

This sentence has been reformulated.

Line 113 the term "extubated alive" is a strange phrase. Would recommend removing "alive". Does this mean patients who were deceased post-re-intubation were excluded? If so, this would potentially skew your results to showing falsely favorable outcomes because the poor outcomes were already removed from analysis, therefore needs to be stated as a major limitation of the study.

We have removing the term “alive”. No, only patients deceased before an first extubation were excluded (never extubated).

The line 122-126 is also awkwardly phrased and need re-writing.

These sentences have been reformulated.

Line 128-131 needs re-writing for clarity. e.g. secondary endpoints included the reintubation rate on day 7 post-extubation,....

These sentences have been reformulated.

In line 220, “in respectively” is a phrase that doen’t make sense grammatically.

This sentence has been reformulated.

Line 222, “at 7th day” also is a strange phrase-better to say “with maintaining successful extubation by post-extubation day 7.”

This sentence has been reformulated.

Line 224-226 was confusingly and the point was lost. Please re-phrase.

These sentences have been reformulated.

Line 237 to 239, it is not enough to say “there is an association” between this and extubation failure but you need to explicitly say and interpret for the reader what the association is actually meaning (you state this in your discussion section but need to write something in the results too), with context of your variables.

These sentences have been reformulated.

2.	In the discussion section, you need to refer to the primary objective which is determining the re-intubation rates with the change in practice but also if the change in practice caused a null, positive, or negative impact on patient care. A lot of the first paragraph are sentences that should be in the result section. The discussion should start by discussing how your main results are meeting or not meeting your objective and the societal or patient impact of your results, such as how you go into this in your second paragraph.

The conclusion has been entirely rewrite following your advices.

Line 271, you wrote “specifically associated specifically”. This sentence needs to be re-written.

This sentence has been rewrite.

Line 273, “the” needs to be inserted before combination.

Modified

Discussion section needs major revising, especially in the context of how your results fit into the current literature especially the two meta-analyses.

Please go through the discussion more thoroughly with significant revision and editing.

The discussion paragraph has been greatly revised integrating the two meta-analyses and edited by an Editing Service.

3.	Conclusion: needs re-writing for clarity and grammar. This sentence suggests a though as derived from the paper, but has to be able to stand alone so that a reader would understand the main point of the paper prior to reading the details in the paper. I think the main point is actually there were no worsened re-intubation rates with the change of practice of increasing the use of HFO and NIV post-extubation in a surgical icu. The wording in the sentence is not only confusing but also misleading. It suggests that using earlier use of non-invasive and HFO will decrease re-intubation, but your study is not a prospective study on the timing of NIV and HFO on reintubation rates.

The conclusion has been entirely rewrite following your advices.
