# Supplementary material for: Safety of changes in the use of noninvasive ventilation and high flow oxygen therapy on reintubation in a surgical intensive care unit: A retrospective cohort study
Source: PLoS One. 2021 Mar 22;16(3):e0249035. doi: 10.1371/journal.pone.0249035 (PMC7984629; doi:10.1371/journal.pone.0249035)
Supplement: Attachment — Submitted filename: reviewer comment #2.docx [file pone.0249035.s008.docx]

1. There needs further revision for clarity of thought and free of grammatical errors. for example, in line 59, "pathophysiology"is not used correctly here. It is a term to describe the physiologic mechanism behind symptoms so should not be used to describe a list of clinical manifestations or symptoms. there should also be a "and" before cardiac dysfunction in line 60. In line 67, the use of the word "curative" is also odd because NIV doesn't "cure" tracheal reintubation, since it is a supportive measure to bridge until the underlying process is addressed so should be referred to as "preventative". In line 69, the phrase "a more recent method" is unclear and refers to that is a more recent developed technology compared to NIV but I think only a reader who would know that context would understand this, so instead of "recent method" should be changed to "a more recently developed technology". But that sentence doesn't clearly distinguish the difference between NIV and HFO. In lines 83 & 84, connect the reason to your unit's change in practice more clearly to the benefits as described in lines 73. Also, it would be more clear to define specific worsening outcomes in line 79, because patient safety should then be addressed as part of your primary objectives and results. In light of the two meta-analyses and studies of prospective randomized controlled studies, at the most your retrospective study can show no worsening of patients but would not be able to prove HFO is superior. In line 109, this sentence "we proceeded to anonymization of database before data analysis" is awkwardly phrased and needs re-writing. Line 113 the term "extubated alive" is a strange phrase. Would recommend removing "alive". Does this mean patients who were deceased post-re-intubation were excluded? If so, this would potentially skew your results to showing falsely favorable outcomes because the poor outcomes were already removed from analysis, therefore needs to be stated as a major limitation of the study. The line 122-126 is also awkwardly phrased and need re-writing. Line 128-131 needs re-writing for clarity. e.g. secondary endpoints included the reintubation rate on day 7 post-extubation,....

In line 220, “in respectively” is a phrase that doen’t make sense grammatically. Line 222, “at 7^th^ day” also is a strange phrase-better to say “with maintaining successful extubation by post-extubation day 7.” Line 224-226 was confusingly and the point was lost. Please re-phrase. Line 237 to 239, it is not enough to say “there is an association” between this and extubation failure but you need to explicitly say and interpret for the reader what the association is actually meaning (you state this in your discussion section but need to write something in the results too), with context of your variables. Line 242, need to add “between” after the word “different.”

2. In the discussion section, you need to refer to the primary objective which is determining the re-intubation rates with the change in practice but also if the change in practice caused a null, positive, or negative impact on patient care. A lot of the first paragraph are sentences that should be in the result section. The discussion should start by discussing how your main results are meeting or not meeting your objective and the societal or patient impact of your results, such as how you go into this in your second paragraph.

Line 271, you wrote “specifically associated specifically”. This sentence needs to be re-written. Line 273, “the” needs to be inserted before combination.

Please go through the discussion more thoroughly with significant revision and editing.

3. Conclusion: needs re-writing for clarity and grammar. This sentence suggests a though as derived from the paper, but has to be able to stand alone so that a reader would understand the main point of the paper prior to reading the details in the paper. I think the main point is actually there were no worsened re-intubation rates with the change of practice of increasing the use of HFO and NIV post-extubation in a surgical icu. The wording in the sentence is not only confusing but also misleading. It suggests that using earlier use of non-invasive and HFO will decrease re-intubation, but your study is not a prospective study on the timing of NIV and HFO on reintubation rates.
